# Redefining Health: The Evolution of Health Ideas from Antiquity to the Era of Value-Based Care

**DOI:** 10.7759/cureus.1018

**Published:** 2017-02-09

**Authors:** Ido Badash, Nicole P Kleinman, Stephanie Barr, Julie Jang, Suraiya Rahman, Brian W Wu

**Affiliations:** 1 Keck School of Medicine, University of Southern California; 2 School of Pharmacy, University of Southern California; 3 Division of Children with Special Needs, Heart of the Ozarks Healthcare Center; 4 Division of Pediatrics, Keck School of Medicine, University of Southern California

**Keywords:** healthcare, value-based care, information technology, world health organization, ancient medicine, defining health, health system, hippocrates, value, patient centered outcomes

## Abstract

The current healthcare system in the United States (US) is characterized by high costs and poor patient outcomes. A value-based healthcare system, centered on providing the highest quality of care for the lowest cost, is the country’s chosen solution for its healthcare crisis. As the US transitions to a value-based model, a new definition of health is necessary to clearly define what constitutes a healthy state. However, such a definition is impossible to develop without a proper understanding of what “health” actually means. To truly understand its meaning, one must have a thorough historical understanding of the changes in the concept of health and how it has evolved to reflect the beliefs and scientific understanding of each time period. Thus, this review summarizes the changes in the definition of health over time in order to provide a context for the definition needed today. We then propose a new definition of health that is specifically tailored to providers working in the era of value-based care.

## Introduction and background

Researchers, policymakers, and healthcare professionals of all political stripes agree that we are in the midst of a “multifactorial and growing crisis of health care systems” [1]. The United States (US) allocates enormous resources to healthcare in return for poor patient outcomes, and the high cost of care has led to severe health disparities between demographic groups [2-3]. The resulting crisis has been met with a push towards a value-based healthcare system, with payment tied to outcomes, that is aimed at reducing societal costs while improving the quality of care to individual patients [4]. As policymakers strive to transform healthcare into a value-based system, there is a parallel process that must occur, where society needs to define very clearly and unambiguously what constitutes health.

## Review

Since the earliest days of mankind, cultures around the world have sought the elusive understanding of what it means to be healthy. This definition has evolved many times, often reflecting the specific beliefs and the levels of scientific and medical understanding of that particular era. Understanding these changes provides a context for the new definition that is needed in the present age.

### Ancient medicine

**Figure 1:**
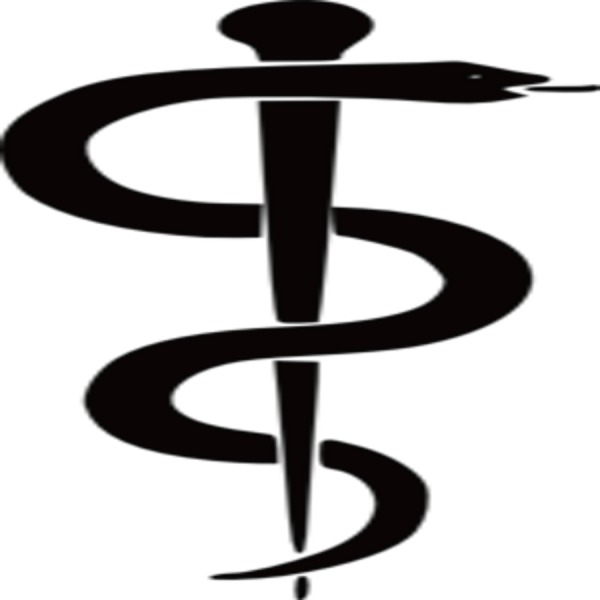
The Rod of Asclepius, an International Symbol of Medicine

In ancient times, health fell largely under the influence of religion and was equivalent to gaining favor with deities. Religious healers believed that in order to achieve health, it was necessary for individuals to pray and sacrifice to the gods in order to propitiate them [5]. In several medical papyri of ancient Egypt, headaches were attributed to the actions of demons and supernatural forces that had to be appeased in order to be cured [6]. In the 11th to 12th century BCE, in ancient Greece, those seeking healing would make pilgrimages to the temples of offended deities in order to appease their wrath. Some would go directly to the temple of the healing god Asclepius, where prayers and sacrifices were performed in exchange for dream cures that came while patients slept [7]. The rod of Asclepius, an international symbol of medicine, is a reminder of humanity’s ancient belief that health was endowed by deities of medicine and healing [8] (Figure 1).

### The Hippocratic concept of health

The first major break from supernatural concepts of health came from the school of Hippocrates around the fifth century BCE. Considered the “Father of Modern Medicine,” Hippocrates was the first to separate Greek medicine from magical and religious beliefs and establish the relationship between environmental/personal cleanliness and the origin of disease [9]. Hippocrates believed that disease resulted from imbalances between four bodily fluids - black bile, yellow bile, phlegm, and blood. Thus, Hippocratic medicine considered health to be a state of bodily balance that could be achieved through behavioral and medicinal actions [10]. While some eastern medical practices still retain elements of spirituality in medicine, all modern health practices in the developed world, including eastern medicine, rely on the Hippocratic concept of health as a product of environmental and behavioral factors.

### Holistic and societal health in the Roman Empire and pre-WHO era

The scientific progress made during the era of the Roman Empire in the first century BCE to fifth century CE further transformed humanity’s understanding of health. The most prominent physician of the Roman Empire, Galen, expanded upon the Hippocratic definition of health by stating that the balance between the four bodily fluids also determined temperament and personality. Keeping with the architectural fervor of the Roman Empire, Galen also felt that a physician needed to study the whole body just as an architect needed to follow a plan [11]. Thus, Galen contributed to the development of a more holistic idea of health that considered the whole patient, including mental and emotional states [12]. Moreover, the construction of fresh water aqueducts, sewer systems, and public bathing houses during the time of the Roman Empire was the first organized effort to maintain health on a population scale. Through Roman innovations in sanitation and public health, the focus of healthcare began to shift from a single individual to an entire society [13].

The discovery of cells (1665 CE), microorganisms (1676 CE), and genes (1866 CE), along with the uncovering of many chemical and molecular entities that keep the human body and mind in equilibrium, further refined the concepts of health put forth by Hippocrates and Galen [14-15]. As additional scientific discoveries were made and healthcare delivery methods improved, new definitions of health were also developed to apply healthcare on a societal scale. Although many definitions of health were developed during this transformation, a discussion of health in the modern era must include what many consider the cornerstone of health definitions - the one developed by the World Health Organization (WHO).

### The WHO definition of health

For nearly 70 years, many healthcare professionals have used the definition of health proposed by the World Health Organization in 1948. The WHO defines health as “*a state of complete physical, mental and social well-being, not merely the absence of infirmity or disease." *This definition was developed in the wake of World War II, when the United Nations created the WHO to spark global health initiatives for individuals all over the world to achieve “the highest possible level of health” [16]. At the time, this was considered a revolutionary way of thinking, as the definition expanded the concept of health from mere concern about the physical characteristics of a disease to a consideration of the social determinants which affect a patient’s health outcomes and quality of life. Additionally, it focused on the need to achieve *well-being*, which differs from the simple lack of disease by also incorporating psychosocial, behavioral, and environmental considerations. The WHO’s new definition of health had enormous social impact in the sense that it challenged political, academic, community, and professional organizations to allocate resources in order to help achieve the lofty goal of universal well-being [16]. Because of its expanded scope, it was considered a positive step forward in the perception and achievement of health [17].

This definition has not been wholly successful, however. Many argue that the well-intentioned purpose of WHO remains unfulfilled due to a variety of issues, including the difficulty of dealing with complex, chronic conditions along with the disparity of resources between the developed and developing world. As an example of its unachieved purpose, authors frequently note that the United Nation’s Millennium Development Goals, a list of common goals for the world community to achieve by 2015, and which included global health initiatives, were not fulfilled on schedule [18-19]. Some pinpoint the underlying problem as being the impracticality of the WHO’s definition of health, noting that while the definition is positive, ambitious, and offers unlimited opportunities for global improvement, it is not practical because it is too fundamental and cannot be reliably and equitably enforced [20-21]. The definition has also been criticized for its conceptual drawbacks: that it is actually a closer description of happiness rather than health. These critics maintain that while health can be considered a human right, arguing for happiness as an equivalent right is more difficult and more vulnerable to subjective opinion [17].

### Alternative definitions of health

In light of some of the shortcomings of the WHO definition, others have come forward with different definitions of health. René Dubos, in his 1959 humanistic book, *The Mirage of Health*, rebelled against the idea that health could be attained by technological means. He instead defined good health as the condition best suited for each individual to reach his or her personal and social goals [22]. Well-known sociologist Abraham Maslow, in his 1968 book, *Towards a Psychology of Being*, wrote that health was based on the fulfilment of needs in a particular order: first physical needs; then safety; love and belonging; esteem; and lastly self-actualization [23]. PI Ahmed, in his 1977 book, *Toward a New Definition of Health: Psychosocial Dimensions, *maintained that health is a relative term that must recognize the specific circumstances of the individual and society [24]. According to this definition, health is not a state that is desirable in itself but as a means towards the fulfillment of role obligations.

Still more alternative definitions of health are being sought in light of the myriad challenges facing not only the United States healthcare system, but also systems in other developed and developing countries. J Bircher, an expert on health economics, proposes that traditional definitions of health and disease must be updated due to the global crisis of healthcare. He argues that the definition of health should be changed to “*a dynamic state of well-being, characterized by an individual’s physical, mental and social potential to meet the demands of life unique to the individual’s age, culture and personal responsibility*” [1]. But in an age where economic considerations are a key component of healthcare disparities and value-based healthcare is becoming the norm, this new definition may not go far enough.

### Moving towards value-based healthcare and a new definition of health

**Figure 2:**
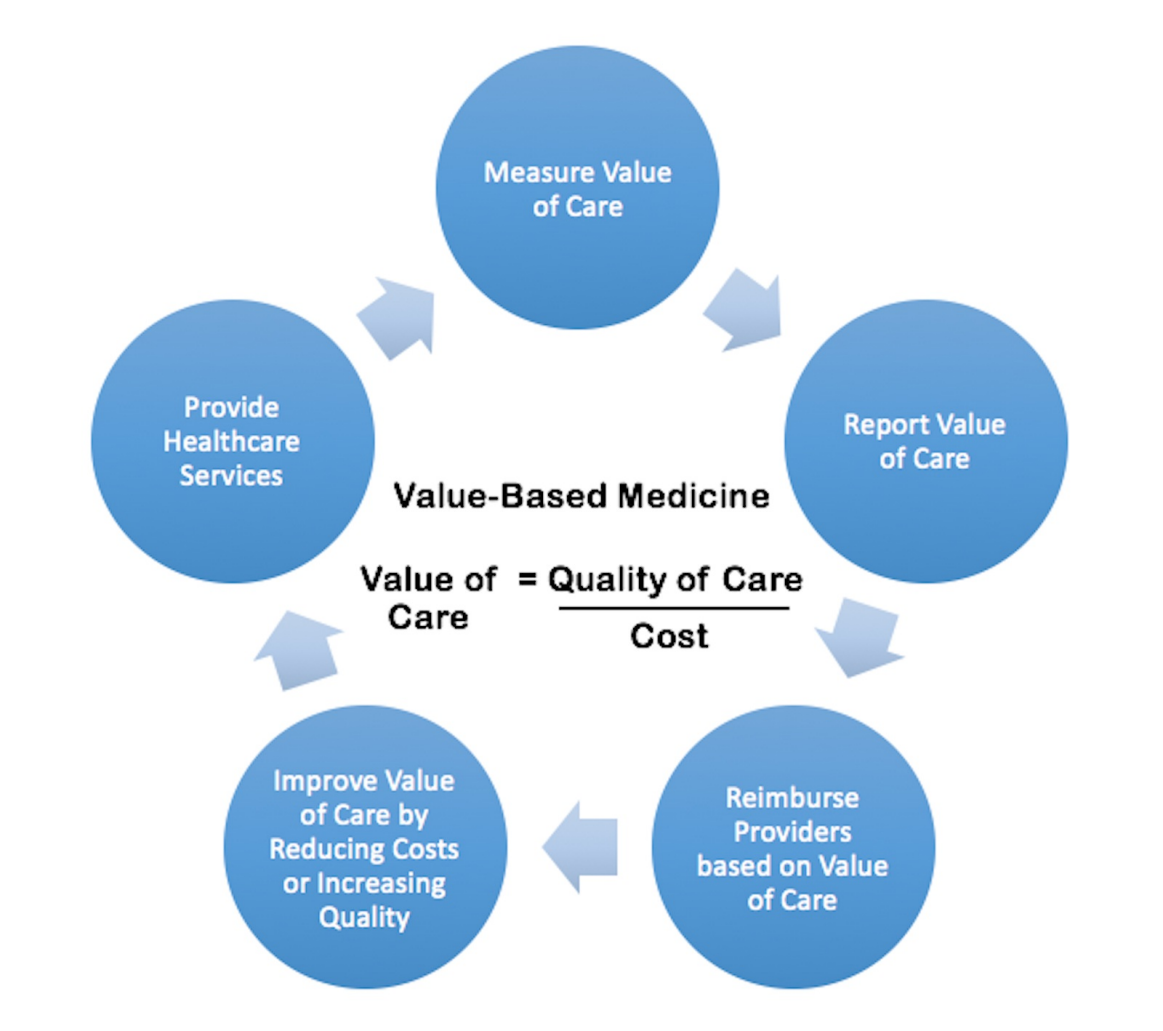
A Model for Value-Based Medicine

After centuries of medical advances, healthcare today is limited more by its systems of delivery and implementation than by the borders of scientific knowledge [25]. In other words, the underlying problem with modern healthcare in the United States is that the costs are high but the quality attained for that cost is unacceptably low. Renowned Harvard economist, Michael Porter, noting these rising costs and the uneven quality of healthcare, observed that the problem is not a lack of diligence, skill, or scientific progress, but the structure of the healthcare delivery system itself. Porter observed that healthcare providers are trying to deliver 21st century medicine with a 19th century market-based delivery system [26]. Simply put, competition in the current volume-based healthcare system does not equate with value for the patient because financial success for providers does not equate with health-related success for the patient. Porter believes that healthcare should instead be value-based, with value defined as the health outcomes achieved per dollars spent [27]. Such a value-based system decisively fastens the connection between reimbursement, cost of service, and the attainment of health outcomes that are valued by patients (Figure 2).

According to Porter, an effective value-based healthcare system should be supported by six different principles. These include: 1) organizing into integrated practice units (IPUs) that provide the full cycle of care for a patient’s given condition, including comorbidities, complications, and other condition-specific needs; 2) measuring costs and outcomes for every patient; 3) moving to bundled payments that cover the full care cycle for acute medical conditions, the overall care for chronic conditions for a pre-determined period, or primary and preventive care for a specific patient population; 4) integrating healthcare delivery across separate facilities by assigning a single physician leader for each patient and adopting common protocols between sites; 5) geographically expanding healthcare providers’ coverage through the use of satellite locations and affiliations with local community providers; and lastly, 6) supporting these changes with a healthcare information technology (IT) platform that is patient-centered, versatile, makes medical data easy to manage, and is accessible to all healthcare professionals [26-28] (Table 1).

**Table 1:** Porter’s Six Principles of an Effective Value-Based Healthcare System

	Principle Summary	Benefits of Action
1	Organize medical services into integrated practice units (IPUs)	IPU personnel from different specialties work together as a team towards the common goal of maximizing the patient’s overall outcomes as efficiently as possible.
2	Measure costs and outcomes	Measuring costs and outcomes along the full cycle of care allows estimation of value of care and provides actionable feedback to healthcare professionals so that they can improve efficiency of care.
3	Move to bundled payments	Bundled payments uncouple payment from service volume to reduce unnecessary spending, and instead tie reimbursement to the overall care for a patient with a particular medical condition.
4	Integrate healthcare between facilities	Integration of healthcare eliminates the division and redundancy of services and enhances the level of healthcare delivered in each location by standardizing quality.
5	Expand geographically with satellite facilities and community affiliations	Geographic expansion allows for the delivery of high quality, integrated healthcare on a local level to reach as many patients as possible within their local communities.
6	Build a suitable information technology (IT) platform	A successful IT platform helps members of an IPU work well together, enables measurement of quality and cost of care, and integrates all parts of a well-structured delivery system.

The pressure is mounting for a transition to a specific value-based system which will both control rising healthcare costs while significantly improving the quality of outcomes for patients. Researchers writing about this change note that accountable care organizations, medical homes, and bundled care products are all different ways of initiating this transition and making wide-scale adoption of value-based care possible [3]. In fact, the United States Secretary of Health and Human Services has recently set a goal of tying 50% of Medicare payments to accountable care organizations and bundled payment arrangements by 2018, furthering the transition to a value-based system [29].

Establishing an effective healthcare system centered around maximizing patient value will also require the efforts of employers, community healthcare providers, and patients themselves. Some employers are already using bundled payment plans to reimburse hospitals for the care of employees [28]. Community healthcare providers may collaborate with larger hospitals in order to extend preventive, mental, and long-term health services to patients in their local communities, where they are more closely surrounded by their families and social networks [27-28]. Finally, the active participation of patients in healthcare is being increasingly utilized through programs like the NIH Precision Medicine Initiative. This program, aimed at extending precision medicine to all diseases by enrolling a large number of patient volunteers in national cohort studies, represents a mechanism for transitioning to a dynamic learning healthcare system that empowers patients by relying on their direct involvement [30]. This integration of hospitals, employers, community programs, and patients provides a platform for maintaining not only patients' physical health, but also their emotional, mental, and social wellness.

**Figure 3:**
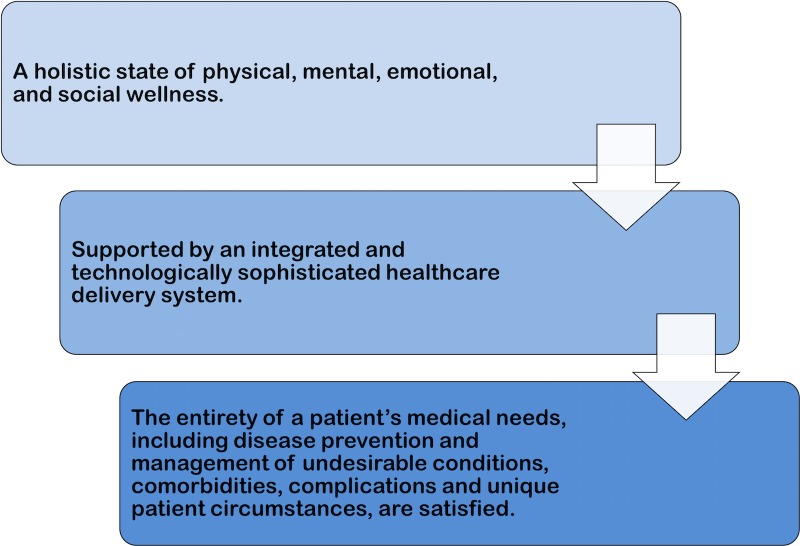
A Schematic View of the Proposed New Definition of Health

If these experiments work, and the transition to a new age of healthcare is successful, a new definition of health is likewise needed. And in this era where value-based medicine is taking off, such a definition of health should be: *a holistic state of physical, mental, emotional, and social wellness supported by an integrated and technologically sophisticated healthcare delivery system tailored to meet the entirety of a patient’s medical needs, including disease prevention and management of undesirable conditions, comorbidities, complications, and unique patient circumstances *(Figure 3). This definition adds to the WHO definition the critical need for technology and infrastructure to support a healthcare system that is centered around patients. Importantly, the definition highlights the importance of satisfying all of a patient’s specific needs, which must encompass the entire spectrum of care including management of comorbidities, complications, and unique patient circumstances, as well as prevention of future illness. By emphasizing patients, their specific medical needs, and the healthcare system that can satisfy these needs, this new definition sets an unambiguous and enforceable standard that providers can strive to achieve.

While the move to a value-based healthcare system is desirable, researchers note that a fundamental problem in the transition is the lack of a clear vision of what form the final product will assume [3]. Much has been written about the principles guiding a value-based healthcare system, but until a specific system is actually designed and described in detail, it remains impossible to fully adopt. Additionally, the reliance of a value-based system on quality of care requires that patient outcomes be measured and compared. It will be challenging to fairly evaluate patient outcomes, given that those outcomes which are considered desirable vary not only by each patient’s unique condition and circumstances, but by the elements of well-being that patients, payers, and society value. 

Expectations in a value-based system must be more complex than merely attaining “positive” outcomes. These expectations further reflect the need for an attainable and flexible definition of health, like the one we propose, that takes into account all of a patient’s unique needs. As medical advances allow us to maintain patient health in ways that were not previously possible, the challenge will be in harnessing this power towards serving the needs of the individual while still maintaining the ability to cover the whole population justly. 

## Conclusions

Societal changes and scientific advances throughout history have brought about enormous improvements in the achievement of health. Today, an optimized level of “health,” whatever the definition might be, is fathomable and achievable if given unlimited resources. The problem lies in that resources are not unlimited, and are in fact disproportionately allocated between demographic groups. A value-based system, designed to provide a high quality of healthcare for the lowest cost, is a solution to the growing crisis of healthcare systems. A major problem with value-based care, however, is that these health outcomes are subjective and determined by individual patient needs and values. A new definition of “health,” which incorporates a description of well-being, specific patient needs, and the organizational, value-based system required to satisfy those needs, is now necessary. The definition we propose will help focus national efforts aimed at improving access to healthcare and installing a value-based system that brings the United States out of its healthcare crisis. 
